# Identifying circRNA-miRNA interaction based on multi-biological interaction fusion

**DOI:** 10.3389/fmicb.2022.987930

**Published:** 2022-12-22

**Authors:** Dunwei Yao, Lidan Nong, Minzhen Qin, Shengbin Wu, Shunhan Yao

**Affiliations:** ^1^Department of Gastroenterology, The People’s Hospital of Baise, Baise, China; ^2^The Southwest Affiliated Hospital of Youjiang Medical University for Nationalities, Baise, China; ^3^Department of Child Healthcare, Baise Maternal and Child Hospital, Baise, China; ^4^Department of Pulmonary and Critical Care Medicine, The People's Hospital of Baise, Baise, China; ^5^Medical College of Guangxi University, Nanning, China

**Keywords:** circRNA-miRNA interaction, multi-biological interaction fusion, inductive matrix completion, network embedding, computational method

## Abstract

CircRNA is a new type of non-coding RNA with a closed loop structure. More and more biological experiments show that circRNA plays important roles in many diseases by regulating the target genes of miRNA. Therefore, correct identification of the potential interaction between circRNA and miRNA not only helps to understand the mechanism of the disease, but also contributes to the diagnosis, treatment, and prognosis of the disease. In this study, we propose a model (IIMCCMA) by using network embedding and matrix completion to predict the potential interaction of circRNA-miRNA. Firstly, the corresponding adjacency matrix is constructed based on the experimentally verified circRNA-miRNA interaction, circRNA-cancer interaction, and miRNA-cancer interaction. Then, the Gaussian kernel function and the cosine function are used to calculate the circRNA Gaussian interaction profile kernel similarity, circRNA functional similarity, miRNA Gaussian interaction profile kernel similarity, and miRNA functional similarity. In order to reduce the influence of noise and redundant information in known interactions, this model uses network embedding to extract the potential feature vectors of circRNA and miRNA, respectively. Finally, an improved inductive matrix completion algorithm based on the feature vectors of circRNA and miRNA is used to identify potential interactions between circRNAs and miRNAs. The 10-fold cross-validation experiment is utilized to prove the predictive ability of the IIMCCMA. The experimental results show that the AUC value and AUPR value of the IIMCCMA model are higher than other state-of-the-art algorithms. In addition, case studies show that the IIMCCMA model can correctly identify the potential interactions between circRNAs and miRNAs.

## 1. Introduction

Different from traditional linear non-coding RNA, circRNA is a new type of non-coding RNA with a closed loop structure (3′ and 5′ in circRNA are connected together; Wilusz and Sharp, 2013; Lan et al., 2015b). The unique molecular structure of circRNA ensures that it cannot be affected by RNA exonuclease. In addition, the expression of circRNA is more stable and not easily degraded than other linear non-coding RNA. Further experiments proved that circRNA is rich in miRNA binding sites, which can act as a miRNA sponge in cells to splice, transcribe, and modify the expression of parental genes (Qu et al., 2015; Rybak-Wolf et al., 2015).

Recent experimental results show that circRNA plays an important role in many diseases. For example, quantitative real-time PCR (qRT-PCR) detection found that circRNA BCRC-3 is low expressed in bladder cancer tissue cells. Moreover, cricRNA BCRC-3 can directly bind to miRNA miR-182-5p, and then act as a sponge for miRNA miR-182-5p to promote the activity of its target genes. Therefore, circRNA BCRC3 can be used as a tumor suppressor to inhibit the proliferation of bladder cancer cells (Xie et al., 2018). The expression of circRNA hsa_circ_0008068 is significantly down-regulated in prostate cancer cells. There are multiple binding sites between the circRNA and the anticancer miRNA miR-145-3p. CircRNA hsa_circ_0008068 can play an anti-cancer role in prostate cancer cells by regulating miR-145-3p and its target gene WISP1. Therefore, circRNA hsa_circ_000806 may be an important target for the diagnosis and treatment of prostate cancer (Zheng et al., 2020).

With the continuous development of high-throughput sequencing technology, more and more circRNA-miRNA-disease interactions have been confirmed. At the same time, a large number of databases have been developed to store the basic information of circRNA and interactions related to circRNA such as circBase (Glažar et al., 2014), circBank (Liu et al., 2019), circad (Rophina et al., 2020), and circR2Cancer (Lan et al., 2020c). As a benchmark database in the circRNA field, the circBase database stores basic information related to circRNA such as the position of circRNA, the genomic length, the spliced sequence length, and the gene symbol (Glažar et al., 2014). circBank is a professional database dedicated to standardizing circRNA naming (Liu et al., 2019). This database not only provides basic information about circRNA, but also names some newly discovered circRNAs uniformly. The Circad database collects 1,388 experimentally verified circRNA-disease interactions from five different species (Homo sapiens, mice, rats, chickens, and wild boars; Rophina et al., 2020). CircR2Cancer is a new database that stores circRNA-cancer interactions. This database not only stores experimentally verified circRNA-cancer interactions but also circRNA-miRNA interactions and miRNA-cancer interactions (Lan et al., 2020c). In addition to storing experimentally verified interactions, the circR2Cancer database also stores basic information about circRNA and diseases.

The emergence of circRNA-related databases provides a data basis for circRNA-related interaction prediction based on computational methods. Compared with traditional biological identification method, the interaction prediction model based on the computational method has higher accuracy and less time consumption. Guo et al. (2022) presented a computational model to predict circRNA-miRNA interactions by using Word2vec, Structural Deep Network Embedding, Convolutional Neural Network, and Deep Neural Network. Qian et al. (2022) proposed a computational model (CMASG) for circRNA-miRNA interactions prediction based on graph neural network and singular value decomposition. It utilized the graph neural network to learn feature representations of nodes and the lightGBM to predict circRNA-miRNA association. Lan et al. (2021b) developed a computational framework (NECMA) to identify interactions between circRNAs and miRNAs by using network embedding. It extracted features of circRNA and miRNA based on network embedding and predict circRNA-miRNA associations based on neighborhood regularization logic matrix decomposition and inner product. He et al. (2022) proposed a computational approach (GCNCMI) to predict the potential interactions between circRNAs and miRNAs based on graph convolutional neural network. It used the graph convolutional neural network to exact the potential interactions of adjacent nodes and then utilized the embedded representations generated by each layer to predict the final score. Qian et al. (2021) introduced a computational framework (CMIVGSD), to predict circRNA-miRNA interaction by using singular value decomposition and graph variational auto-encoders. Yu et al. (2022) proposed a computational model (SGCNCMI) to identify circRNA-miRNA interactions by combining multimodal information and graph convolutional neural network. Wang et al. (2022) presented a computing method (KGDCMI) to predict the interactions between circRNA and miRNA based on multi-source information fusion. It exacts RNA attribute information from sequence and similarity and captures the behavior information in RNA association based on graph-embedding algorithm. Then, the principal component analysis is used to obtain feature vector, and further the deep neural network is utilized to identify potential circRNA-miRNA interactions. Fang and Lei (2019) fused circRNA-miRNA interaction network, circRNA functional similarity network, and miRNA functional similarity network to construct a circRNA-miRNA heterogeneous network. Then use the K-nearest neighbor algorithm based on restart random walk to predict the potential interaction of circRNA and miRNA.

In this paper, we propose a circRNA-miRNA interaction prediction model (IIMCCMA) based on multi-biological interaction data. This model uses experimentally verified circRNA-miRNA interaction, circRNA-cancer interaction, and miRNA-cancer interaction to construct circRNA-miRNA adjacency matrix, circRNA-cancer adjacency matrix, and miRNA-cancer adjacency matrix, respectively. On the basis of the above adjacency matrix, this model uses Gaussian kernel function and cosine function to calculate circRNA GIP kernel similarity and circRNA functional similarity, as well as miRNA GIP kernel similarity and miRNA functional similarity. In order to reduce the negative impact of noise or redundant information in the known circRNA-miRNA interaction on the prediction model, the IIMCCMA model first uses the known circRNA-miRNA interaction to construct a heterogeneous network. Then we use the network embedding algorithm to extract the potential feature vectors of circRNA and miRNA in heterogeneous networks. In order to make full use of the information contained in different data sources, this model uses a feature fusion method to integrate the similarity features and topological features of entities in the interaction network to form circRNA fusion features and miRNA fusion features, respectively. Finally, on the basis of circRNA fusion features and miRNA fusion features, an improved inductive matrix completion algorithm is used to predict the potential interaction of circRNA and miRNA. The 10-fold cross-validation experiment was used to evaluate the predictive performance of the IIMCCMA model. The experimental results show that the IIMCCMA model achieves better performance than other advanced interaction prediction models. In addition, the case study results show that the IIMCCMA model can correctly identify the potential interaction between circRNA and miRNA.

## 2. Materials and methods

### 2.1 Materials

We use two datasets as gold standard set inhere which is downloaded from circR2Cancer (Lan et al., 2020c) and KGNACDA (Lan et al., 2022a). In dataset 1, there are 756 interactions between 514 circRNAs and 461 miRNAs, 647 interactions between 514 circRNAs and 62 cancers, and 732 interactions between 461 miRNAs and 62 cancers. In dataset 2, there are 330 circRNAs, 79 diseases and 245 miRNAs, 346 circRNA-disease interactions, 146 circRNA-miRNA interactions, and 106 miRNA-disease interactions. Further, we construct an adjacency matrix to represent the above-mentioned interaction network. The adjacency matrix *CM* represents the circRNA-miRNA interactions. If circRNA $CM_{i}$ is related to miRNA $CM_{j}$ , CM(i,j)=1 , otherwise, CM(i,j)=0 . Similarly, the adjacency matrix *CC* represents the circRNA-cancer interactions. If circRNA $CC_{i}$ is related to cancer $CC_{j}$ , CC(i,j)=1 , otherwise, CC(i,j)=0 . The adjacency matrix *MC* represents the miRNA-cancer interactions. If miRNA is related to cancer $MC_{j}$ , $MC\left(i,j\right)=1$ , otherwise, $MC\left(i,j\right)=0$ .

### 2.2 circRNA and miRNA similarity calculation

Based on the assumption that circRNAs with similar functions are often associated with similar miRNAs (Lan et al., 2020a, 2021a, 2022c), circRNA GIP kernel similarity and miRNA GIP kernel similarity are calculated based on the circRNA-miRNA interaction network, respectively. We define *GCS* to represent the Gaussian interaction profile kernel similarity network of circRNA.

The definition of GIP kernel similarity between circRNA $c_{i}$ and circRNA $c_{j}$ is as follows:


$$GCS\left(c_{i},c_{j}\right)=\exp\left(-\gamma_{cs}CM\left(i,:\right)-CM{\left(j,:\right)}^{2}\right)$$

$$\gamma_{cs}=1/\left(\frac{1}{n_{circ}}\overset{n_{circ}}{\underset{i=1}{\sum }}CM{\left(i,:\right)}^{2}\right)$$

where, CM(i,:) represents the *i-th* row of the circRNA-miRNA interaction network *CM*. $n_{circ}$ represents the number of rows of the interaction network *CM.* $\gamma_{cs}$ represents the kernel bandwidth.

Similarly, we define *GMS* to represent the Gaussian interaction profile kernel similarity network of miRNA. The definition of GIP kernel similarity between miRNA $m_{i}$ and miRNA $m_{j}$ is as follows:


$$GMS\left(m_{i},m_{j}\right)=\exp\left(-\gamma_{ms}CM\left(:,i\right)-CM{\left(:,j\right)}^{2}\right)$$

$$\gamma_{ms}=1/\left(\frac{1}{n_{mi}}\overset{n_{mi}}{\underset{i=1}{\sum }}CM{\left(:,i\right)}^{2}\right)$$

where, CM(:,i) represents the *i-th* column of the circRNA-miRNA interaction network *CM*. $n_{mi}$ represents the number of columns of the interaction network *CM.* $\gamma_{ms}$ represents the kernel bandwidth.

In addition, we also use the cosine function to calculate the circRNA functional similarity and the miRNA functional similarity on the basis of circRNA-cancer interaction network and miRNA-cancer interaction network. The cosine similarity measures the similarity between two vectors by the angle between two vectors in a two-dimensional space. If the two vectors point in the same direction, it means that the two vectors are more similar, otherwise, the similarity is lower. Therefore, according to the above cosine similarity theory, the circRNA functional similarity and miRNA functional similarity are defined as follows:


$$CCS\left(c_{i},c_{j}\right)=\frac{\overset{n_{circ}}{\underset{i=1}{\sum }}CC\left(i,:\right)\times CC\left(j,:\right)}{\sqrt{\overset{n_{circ}}{\underset{i=1}{\sum }}CC{\left(i,:\right)}^{2}}\times \sqrt{\overset{n_{circ}}{\underset{i=1}{\sum }}CC{\left(j,:\right)}^{2}}}$$

$$CMS\left(m_{i},m_{j}\right)=\frac{\overset{n_{mi}}{\underset{i=1}{\sum }}MC\left(i,:\right)\times MC\left(j,:\right)}{\sqrt{\overset{n_{mi}}{\underset{i=1}{\sum }}MC{\left(i,:\right)}^{2}}\times \sqrt{\overset{n_{mi}}{\underset{i=1}{\sum }}MC{\left(j,:\right)}^{2}}}$$

where *CCS* and *CMS* represent the circRNA functional similarity network and the miRNA functional similarity network, respectively. $CCS\left(c_{i},c_{j}\right)$ represents the functional similarity between circRNA $c_{i}$ and circRNA $c_{j}$ . CC(i,:) represents the *i-th* row in the circRNA-cancer network *CC*. $n_{circ}$ represents the number of rows in the network *CC*. $\;CMS\left(m_{i},m_{j}\right)$ represents the functional similarity between miRNA $m_{i}$ and miRNA $m_{i}$ . MC(i,:) represents the *i-th* row in the miRNA-cancer network *MC*. $n_{mi}$ represents the number of rows in the network *MC*.

In order to make better use of the circRNA and the miRNA similarity characteristics, we integrate the above two similarities to obtain the circRNA similarity $circ_{sim}$ and miRNA similarity $mi_{sim}$ , which are defined as follows:


$$circ_{sim}\left(c_{i},c_{j}\right)=\{\begin{array}{c} GCS\left(c_{i},c_{j}\right),if\;CCS\left(c_{i},c_{j}\right)=0\\ CCS\left(c_{i},c_{j}\right),otherwise \end{array}$$

$$mi_{sim}\left(m_{i},m_{j}\right)=\{\begin{array}{c} GMS\left(m_{i},m_{j}\right),if\;CMS\left(m_{i},m_{j}\right)=0\\ CMS\left(m_{i},m_{j}\right),otherwise \end{array}$$

where $circ_{sim}\left(c_{i},c_{j}\right)$ represents the integrated similarity between circRNA $c_{i}$ and circRNA $c_{j}$ . $\;mi_{sim}\left(m_{i},m_{j}\right)$ represents the integrated similarity between miRNA $m_{i}$ and miRNA $m_{j}$ . *GCS* represents circRNA GIP kernel similarity. *CCS* represents the circRNA functional similarity. In the same way, *GMS* represents miRNA GIP kernel similarity. *CMS* represents the miRNA functional similarity.

### 2.3 Potential feature extraction and fusion of circRNA and miRNA

In order to reduce the influence of noise or redundant information in the known circRNA-miRNA interaction network, we construct the heterogeneous network $H_{circ-mi}$ . The heterogeneous network is composed of the circRNA-miRNA interaction adjacency matrix *CM* and the transposed matrix $CM^{T}$ of the circRNA-miRNA adjacency matrix. It is defined as follows:


$$H_{circ-mi}=\left[\begin{array}{cc} 0 & CM\\ CM^{T} & 0 \end{array}\right]$$

After obtaining the heterogeneous network, the NetMF algorithm (Qiu et al., 2018) is used to obtain the circRNA-miRNA latent feature matrix with size equals to (m+n)×d . Among them, m represents the number of circRNA in the heterogeneous network and $H_{circ-mi}$ . n represents the number of miRNAs. 𝑑 represents the dimensions of circRNA and miRNA low-dimensional space vectors. Experiments have verified that the model has the best prediction effect when the dimension of the low-dimensional space vector of circRNA and miRNA is set to 16.

In order to make full use of the information of different interaction, we use a fusion method to fuse the circRNA and miRNA topological features ( $circ_{Net}$ , $mi_{Net}$ ) obtained through the NetMF algorithm with the integrated circRNA similarity features and miRNA similarity features, respectively. The fused information can not only describe the characteristics of different data sources, but also describe the complex relationship between circRNA and miRNA more comprehensively. The fusion feature of circRNA circ_feature and the fusion feature of miRNA mi_feature are defined as follows:


$$circ\_f=\left[circ_{Net},circ_{sim}\right]$$

$$mi\_f=\left[mi_{Net},mi_{sim}\right]$$

where $circ_{Net}$ and $mi_{Net}$ represent the topological characteristics of circRNA and miRNA based on the NetMF algorithm, respectively. $circ_{sim}$ and $mi_{sim}$ represents the circRNA integrated similarity and miRNA integrated similarity, respectively.

### 2.4 Prediction of potential interaction between circRNA and miRNA

**Figure 1:**
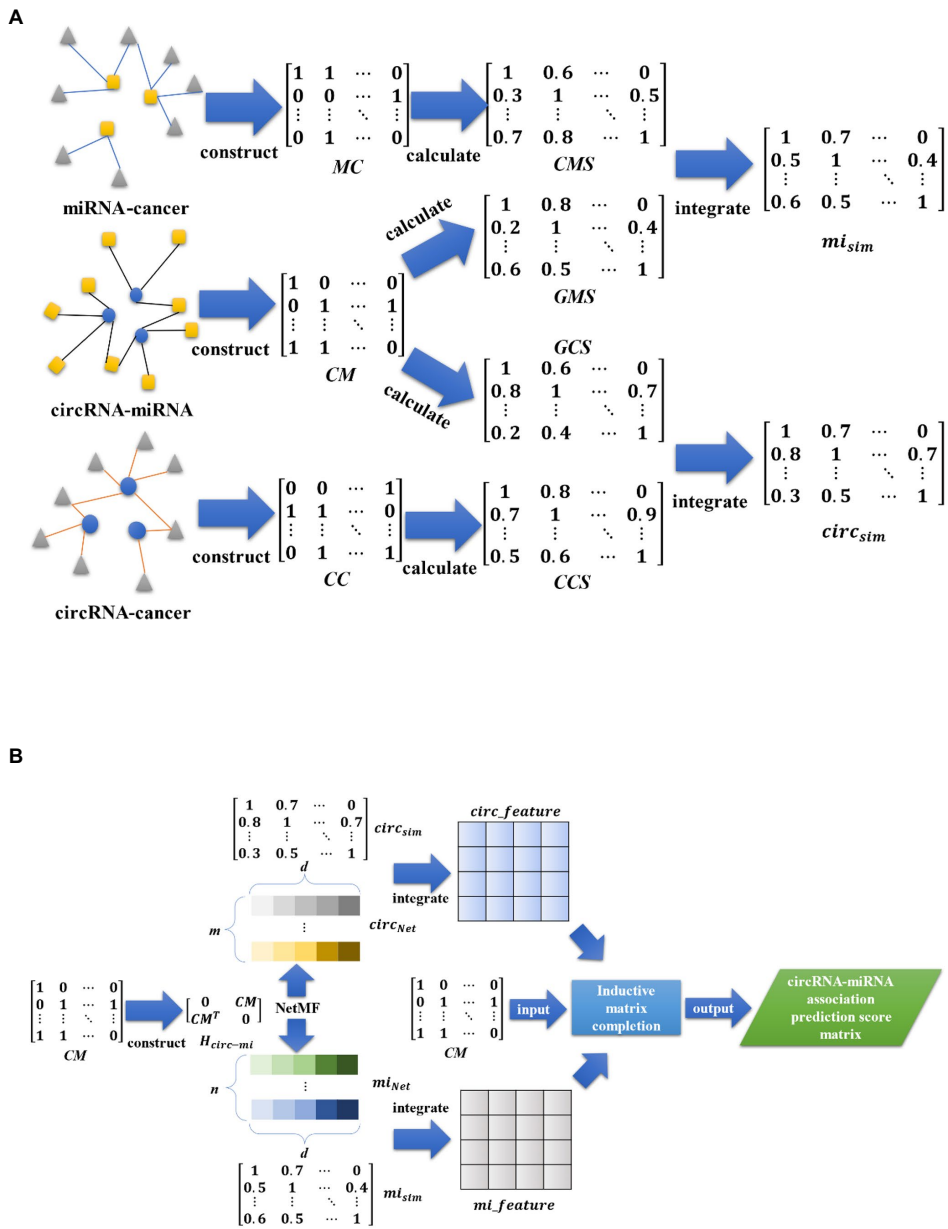
**(A)** Overview of interaction prediction model for circRNA and miRNA based on multi-biological interaction (1). **(A)** Mainly shows the construction of the incidence matrix, the calculation of similarity, and the fusion of similarity. **(B)** Overview of interaction prediction model for circRNA and miRNA based on multi-biological interaction (2). **(B)** Mainly shows the construction of heterogeneous networks, feature extraction based on NetMF algorithm, feature fusion, and calculation of interaction prediction scores.

In this paper, we propose a circRNA-miRNA interaction prediction model (IIMCCMA) based on an improved inductive matrix completion algorithm. This model is implemented based on the known circRNA-miRNA interaction, the fusion feature of circRNA and the fusion feature of miRNA. The specific implementation process of the IIMCCMA model is shown in Figures 1A,B.

Many studies have found that the sparsity problem of biological interaction networks is very serious. Taking the circRNA-miRNA interaction network used in this paper as an example, the circRNA-miRNA interaction network *CM* is composed of 756 interactions between 514 circRNAs and 461 miRNAs. Obviously, the interaction network *CM* is very sparse (the matrix density is 0.0032). In addition, in the calculation process of the inductive matrix completion algorithm (Jain and Dhillon, 2013; Lan et al., 2015a; Si et al., 2016; Nazarov et al., 2018), due to the high sparsity of the known interaction matrix, a relatively large amount of effective information will be lost in the process of low-dimensional mapping, which will affect the prediction effect of the circRNA-miRNA potential interaction prediction model. Therefore, in order to alleviate the negative impact of the high sparsity of the interaction network on the model, we modify the mapping method of the low-rank matrix in the inductive matrix completion algorithm. Specifically, in order to better protect the structural information in the sparse matrix, we perform multiple low-dimensional mapping operations to obtain multiple low-rank matrices with different dimensions. Then we use low-rank matrices of different dimensions to calculate the potential interaction prediction scores of circRNA and miRNA. Finally, the prediction score matrix calculated from the low-rank matrix of different dimensions is integrated to realize the potential interaction prediction of circRNA and miRNA.

In summary, the objective function of the circRNA-miRNA potential interaction prediction model based on the fusion feature and the improved inductive matrix completion algorithm is as follows:


$$\underset{W,H}{\min}\frac{1}{2}∥CM-CM_{Pre}{∥}_{F}^{2}+\frac{\theta_{1}}{2}∥W_{i}^{d_{i}}{∥}_{F}^{2}+\frac{\theta_{2}}{2}∥H_{i}^{d_{i}}{∥}_{F}^{2},W_{i}^{d_{i}}\geq 0,H_{i}^{d_{i}}\geq 0$$

$$CM_{Pre}=mi\_fW_{i}^{d_{i}}H_{i}^{d_{i}}{}^{T}circ\_f^{T}$$

where 𝐶𝑀 represents the known circRNA-miRNA interaction matrix. $CM_{pre}$ represents the predicted circRNA-miRNA interaction matrix. $W_{i}^{d_{i}}$ and $H_{i}^{d_{i}}$ represent the 𝑑-dimensional low-rank matrix obtained through the *i-th* complete iteration of the circRNA-miRNA interaction matrix. 𝜃1 and 𝜃2 represent the regularization parameters. According to the previous research, we set $\theta_{1}=\theta_{2}=1∥.∥F$ represents the Frobenius norm of the matrix (F-norm). $\frac{\theta_{1}}{2}∥W_{i}^{d_{i}}{∥}_{F}^{2}$ and $\frac{\theta_{2}}{2}∥H_{i}^{d_{i}}{∥}_{F}^{2}$ are used to prevent overfitting. In order to find the minimum value of the objective function, we first set up the random dense matrices of $W_{i}^{d_{i}}$ and $H_{i}^{d_{i}}$ , and then update the matrices $W_{i}^{d_{i}}$ and $H_{i}^{d_{i}}$ through iterative equations. When the convergence condition is met, we will stop iteration. The iterative equation are defined as follows:


$$W_{i}^{d_{i}}\leftarrow W_{i}^{d_{i}}\frac{mi\_f^{T}*CM*circ\_fH_{ini}}{mi\_f^{T}mi\_fW_{ini}H_{ini}{}^{T}circ\_f^{T}circ\_fH_{ini}+\theta_{1}W_{ini}}$$

$$H_{i}^{d_{i}}\leftarrow H_{i}^{d_{i}}\frac{\begin{array}{l} circ\_feature^{T}\\ CM^{T}mi\_featureW_{ini} \end{array}}{\begin{array}{l} circ\_feature^{T}circ\_featureH_{ini}W_{ini}{}^{T}mi\_feature^{T}\\ mi\_featureW_{ini}+\theta_{2}H_{ini} \end{array}}$$

where circ_feature and mi_feature represent the fusion characteristics of circRNA and the fusion characteristics of miRNA, respectively. $circ\_feature^{T}$ and $mi\_feature^{T}$ represent the transposition matrix of the circRNA fusion feature matrix and the transposition matrix of the miRNA fusion feature matrix, respectively. $CM^{T}$ represents the transposed matrix of the known circRNA-miRNA interaction matrix. $W_{ini}$ and $H_{ini}$ represent the initial random dense matrix of the low-rank matrix $W_{i}^{d_{i}}$ and $H_{i}^{d_{i}}$ , respectively.

Finally, the calculation method of the circRNA and miRNA correlation prediction score matrix is defined as follows:


$$Pre_{circRNA-miRNA}=\frac{\sum_{i}^{k}mi\_featureW_{i}^{d_{i}}H_{i}^{d_{i}}{}^{T}circ\_feature}{k}$$

where $Pre_{circRNA-miRNA}$ represents the final circRNA-miRNA potential interaction prediction score matrix. Each item in the matrix represents the interaction probability score between circRNA and miRNA. The higher the score, the greater the probability that there is an exact interaction between circRNA and miRNA. 𝑘 indicates the number of complete iterations of the iterative equation. The best prediction performance is obtained when 𝑘 = 2 and $d_{1}$ = 128, $d_{2}$ = 64.

### 2.5 Performance evaluation

In order to evaluate the performance of model in predicting the potential interaction between circRNA and miRNA, the 10-fold cross-validation experiment is used to evaluate the performance. In 10-fold cross-validation, the known circRNA-miRNA interactions are randomly divided into 10 subsets. Then, in each round of cross-validation experiments, nine subsets are taken from 10 subsets as the training set for model training and the remaining subset is used as the test set. The final interaction prediction score of circRNA and miRNA is obtained. The higher the score, the higher the probability that there is a biological interaction between circRNA and miRNA. Afterward, we ranked the interaction prediction scores between circRNA and miRNA in descending order. Then, the true positive rate (*TPR*) and false positive rate (*FPR*) are calculated by modifying the threshold. The calculation of *TPR* and *FPR* are defined as follows:


$$TPR=\frac{TP}{TP+FN}$$

$$FPR=\frac{FP}{FP+TN}$$

Finally, a receiver operating curve (ROC) based on the true positive rate and false positive rate is plotted, and the area under the ROC curve (AUROC value) is calculated to evaluate the predictive ability of the model. Similarly, the area of the curve (AUPR value) based on *precision* and *recall* is also used to evaluate the performance of the predictive model. The calculation of *precision* and *recall* is defined as follows:


$$Precision=\frac{TP}{TP+FP}$$

$$Recall=\frac{TP}{TP+FN}$$

where TP means that the classifier predicts the number of positive samples in the actual positive samples. FP represents the number of positive samples is predicted in the actual negative samples. TN means that the classifier predicts the number of negative samples in the actual negative samples. FN indicates the number of actual positive samples that are predicted to be negative.

## 3. Results and discussion

### 3.1 Compare with other models

**Figure 2:**
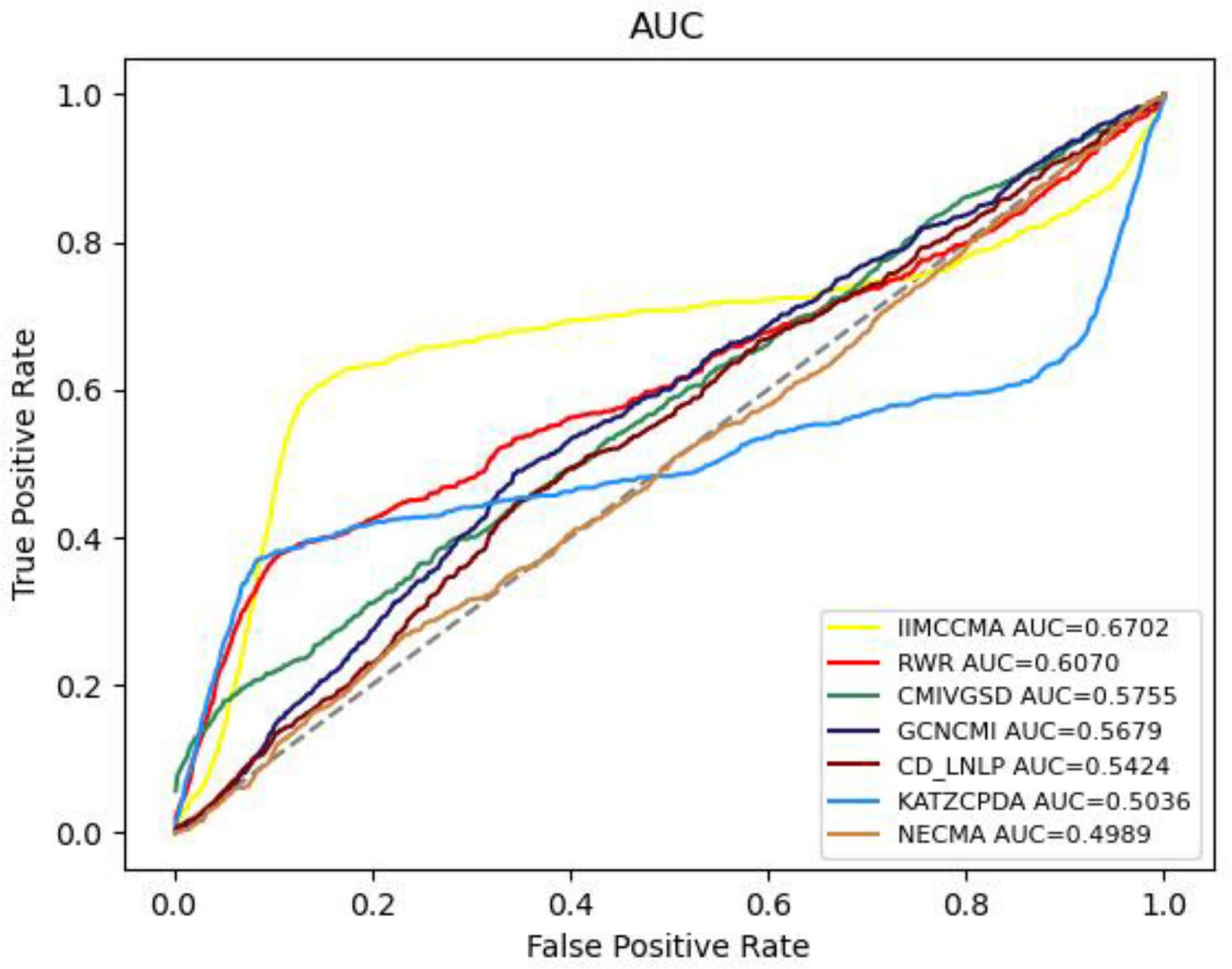
Performance comparison between IIMCCMA and other models on dataset 1 in term of AUROC.

In order to further demonstrate the performance of IIMCCMA, we compare it with the other six prediction methods (NECMA; Lan et al., 2021b, GCNCMI; He et al., 2022, CMIVGSD; Qian et al., 2021, CCD-LNLP; Zhang et al., 2019, RWR; Vural et al., 2019, and KATZCPDA; Fan et al., 2018). As shown in Figure 2, under the 10-fold cross-validation experiment on dataset 1, the AUROC value of NECMA is 0.4898, the AUROC value of CMIVGSD is 0.5755, the AUROC value of GCNCMI is 0.5679, the AUROC value of CD-LNLP is 0.5424, the AUROC value of RWR is 0.6070, the AUROC value of KATZCPDA is 0.5036, and the AUROC value of IIMCCMA is 0.6702. Therefore, from the experimental results, it can be found that the IIMCCMA model has a higher AUROC value than other interaction prediction models on dataset 1.

**Figure 3:**
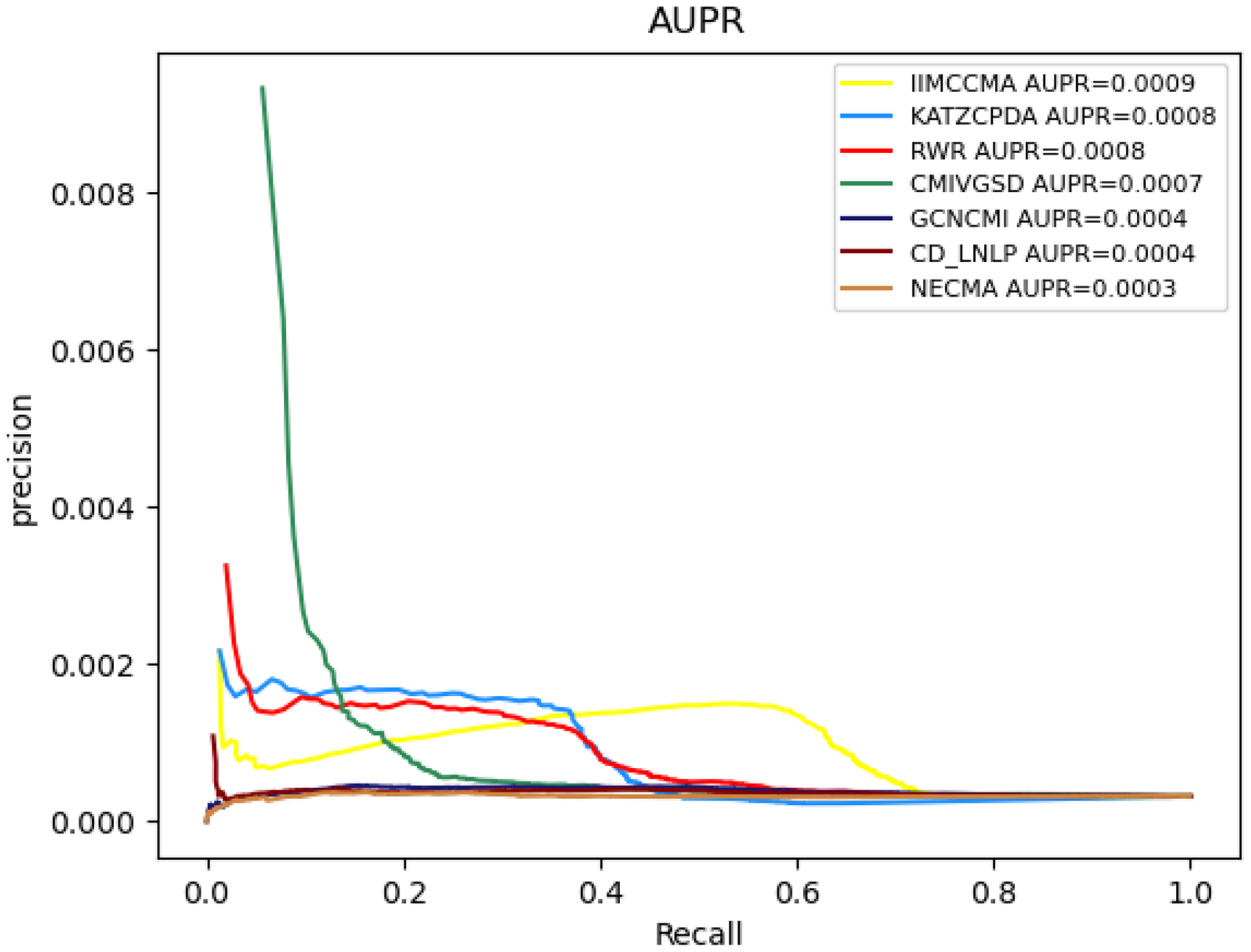
Performance comparison between IIMCCMA and other models on dataset 1 in term of AUPR.

As shown in Figure 3, under the 10-fold cross-validation experiment on dataset 1, the AUROC value of NECMA is 0.0003, the AUPR value of CMIVGSD is 0.0007, the AUPR value of GCNCMI is 0.0004, the AUPR value of CD-LNLP is 0.0004, the AUPR value of RWR is 0.0008, the AUPR value of KATZCPDA is 0.0008, and the AUPR value of the IIMCCMA model is 0.0009. It can be found from the experimental results that the IIMCCMA model achieves a higher AUPR value than the other models on dataset 1.

**Figure 4:**
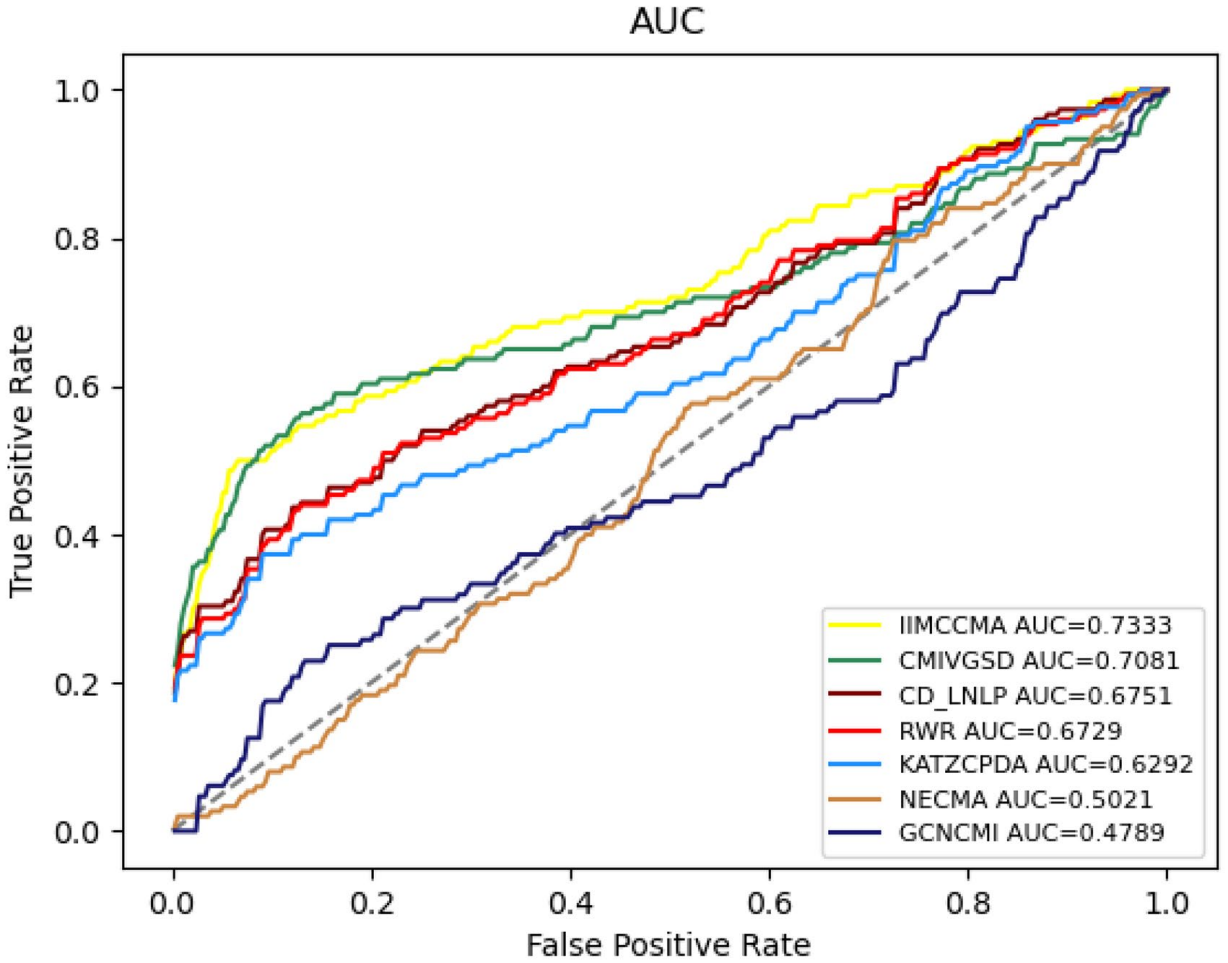
Performance comparison between IIMCCMA and other models on dataset 2 in term of AUROC.

The Figure 4 shows the performance comparison in term of AUROC on dataset 2. It can be found that the AUROC value of NECMA is 0.5021, the AUROC value of CMIVGSD is 0.7081, the AUROC value of GCNCMI is 0.4789, the AUROC value of CD-LNLP is 0.6751, the AUROC value of RWR is 0.6729, the AUROC value of KATZCPDA is 0.6292, and the AUROC value of IIMCCMA is 0.7333. It demonstrates that IIMCCMA outperforms than other prediction models on dataset 2.

**Figure 5:**
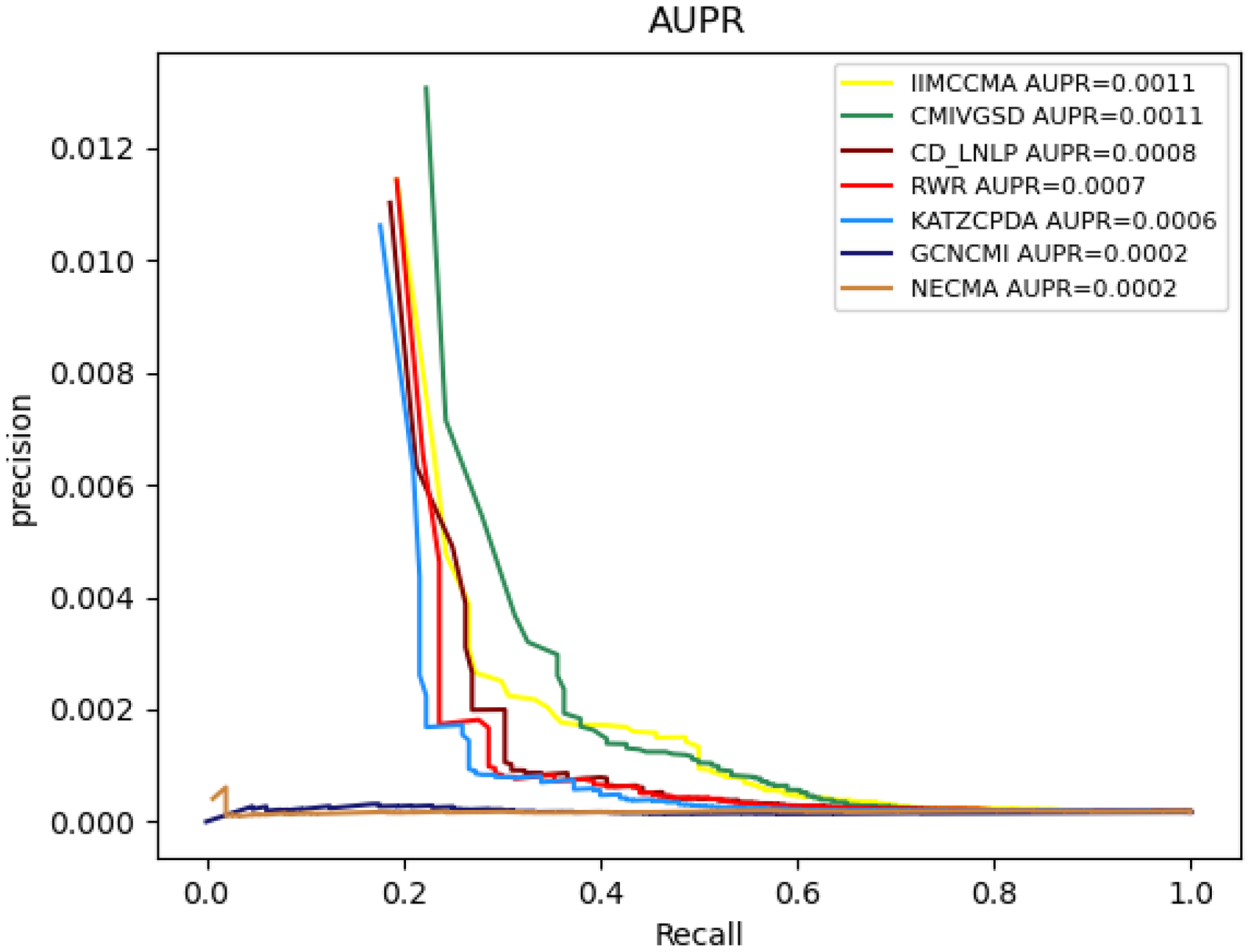
Performance comparison between IIMCCMA and other models on dataset 2 in term of AUPR.

As shown in Figure 5, the AUPR value of NECMA is 0.0002, the AUPR value of CMIVGSD is 0.0011, the AUPR value of GCNCMI is 0.0002, the AUPR value of CD-LNLP is 0.0008, the AUPR value of RWR is 0.0007, the AUPR value of KATZCPDA is 0.0006, and the AUPR value of the IIMCCMA model is 0.0011. It can be found that the IIMCCMA model achieves a higher AUPR value than the other models on dataset 2. In conclusion, under the 10-fold cross-validation experiment, we can find that theIIMCCMA has achieved higher AUROC and AUPR values than the other prediction models. Thus, it can be proved that theIIMCCMA performs better in the potential circRNA-miRNA interactions identification.

### 3.2 Ablation experiment

**Figure 6:**
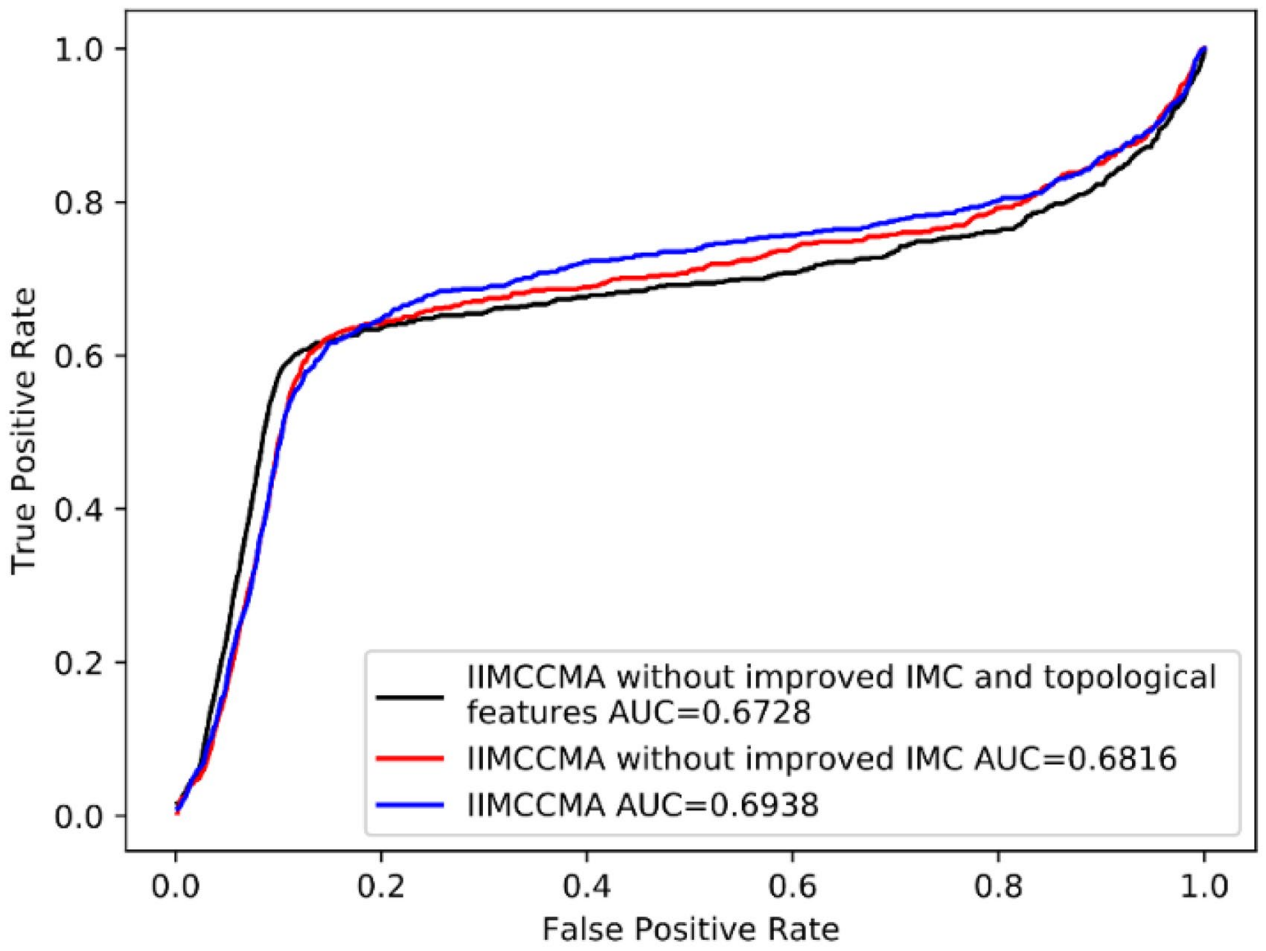
Performance comparison between IIMCCMA model and benchmark model.

In order to verify the effectiveness of the improvements of IIMCCMA, we conduct ablation experiment on dataset 1: CircRNA-miRNA potential interaction prediction model based on multi-source similarity and inductive matrix completion (IIMCCMA without improved IMC and topological features). CircRNA-miRNA potential interaction prediction model based on fusion features and inductive matrix completion (IIMCCMA without improved IMC). We adopt the 10-fold cross-validation experiment and use the AUROC value as the evaluation metrics. As shown in Figure 6, the AUROC value of the circRNA-miRNA potential interaction prediction model based on multi-source similarity (IIMCCMA without improved IMC and topological features) is 0.6728. The AUROC value of the circRNA-miRNA potential interaction prediction model based on fusion features (IIMCCMA without improved IMC) is 0.6816. The AUROC value of IIMCCMA is 0.6938. In summary, based on the original inductive matrix completion algorithm, fusion of similarity features and topological features can improve the predictive ability of the model. Adding improved inductive matrix completion on the basis of fusion features can further improve the performance of the prediction model.

### 3.3 Case study

In order to prove the ability of the circRNA-miRNA potential interaction model (IIMCCMA) based on the multi-source biological interaction data to identify the potential interaction between circRNA and miRNA. This paper builds a case study based on miRNA miR-145-5p. Finally, this paper selects the top 10 circRNAs predicted by the IIMCCMA model that are related to miRNA miR-145-5p, and manually searches the existing literature to prove their relevance.

The top 10 circRNAs related to miRNA miR-145-5p predicted by the IIMCCMA model are shown in Table 1. From Table 1, 10 circRNAs related to miRNA miR-145-5p (hsa_circ_0058063, hsa_circRNA_101981, hsa_circRNA_091420, hsa_circ_100242, circPTN, circPVT1, hsa_circRNA_101996, circCEP128, hsa_circ_0003855, and hsa_circ_0001955) have been confirmed by existing literature. Specifically, the first circRNA hsa_circ_0058063 can be used as the sponge of miRNA miR-145-5p to regulate the expression of miRNA target gene CDK6 and promote the development of bladder cancer (Sun et al., 2019a). In prostate cancer cells, the expression pattern of the second-ranked circRNA hsa_circRNA_101981 was significantly down-regulated. Further experiments showed that miRNA miR-145-5p can regulate the expression of circRNA hsa_circRNA_101981 (He et al., 2018). The expression pattern of the third-ranked circRNA hsa_circRNA_091420 in prostate cancer cells was significantly upregulated. Overexpressed miRNA miR-145-5p can inhibit the expression of circRNA hsa_circRNA_091420 (He et al., 2018). Experimental results show that the fourth-ranked circRNA hsa_circ_100242 can interact with miRNA miR145-5p in bladder cancer cells (Zhu et al., 2020). Experiments show that the fifth-ranked circRNA circPTN is overexpressed in glioma cells and tissues. Further experiments showed that circRNA circPTN can spongy miRNA miR-145-5p and thus play a carcinogenic effect in glioma cells (Chen et al., 2019). CircRNA circPVT1, ranked sixth, was significantly up-regulated in lung adenocarcinoma cells. Experiments show that in lung adenocarcinoma cells, circRNA circPVT1 can be used as an ceRNA for miRNA miR145-5p (Zheng and Xu, 2020). Experiments show that the seventh-ranked circRNA hsa_circRNA_101996 can interact with miRNA miR-145-5p in prostate cancer cells. In addition, overexpressed miRNA miR-1455p can inhibit the expression of circRNA hsa_circRNA_101996 (He et al., 2018). The eighth-ranked circRNA circCEP128 can promote the development of bladder cancer by regulating miRNA miR-145-5p and miRNA’s target gene MYD88 (Sun et al., 2019b). The expression pattern of circRNA hsa_circ_0003855, ranked ninth, was significantly increased in gastric cancer cells. Experimental results show that circRNA hsa_circ_0003855 can take on the sponge effect of miRNA miR-145-5p to promote the proliferation and migration of gastric cancer cells (Zhang et al., 2020). The tenth-ranked circRNA hsa_circ_0001955 can assume the role of miRNA miR-145-5p sponge. Additionally, the downregulated circRNA hsa_circ_0001955 can inhibit the growth of hepatocellular carcinoma tumors (Yao et al., 2019). In summary, through the case study results based on miRNA miR-145-5p, it can be found that the IIMCCMA model can correctly identify the potential biological interaction between circRNA and miRNA.

**Table 1:** Case study based on microRNA miR-145-5p.

Rank	CircRNA	Evidence	Reference
1	hsa_circ_0058063	PMID: 30362519	Sun et al. (2019a)
2	hsa_circRNA_101981	PMID: 30136305	He et al. (2018)
3	hsa_circRNA_091420	PMID: 30136305	He et al. (2018)
4	hsa_circ_100242	PMID: 32218853	Zhu et al. (2020)
5	circPTN	PMID: 31511040	Chen et al. (2019)
6	circPVT1	PMID: 31986409	Zheng and Xu (2020)
7	hsa_circRNA_101996	PMID: 30136305	He et al. (2018)
8	circCEP128	PMID: 30939216	Sun et al. (2019b)
9	hsa_circ_0003855	PMID: 31776711	Zhang et al. (2020)
10	hsa_circ_0001955	PMID: 31822654	Yao et al. (2019)

## 4. Conclusion

Experiments show that circRNA can play an important role in cancer as a miRNA sponge. Therefore, correct identification of the interaction between circRNA and miRNA not only helps to understand the complex disease mechanism, but also contributes to the diagnosis, treatment and prognosis of the disease. Based on circRNA-miRNA interaction, circRNA-cancer interaction and miRNA-cancer interaction, this paper proposes a circRNA-miRNA potential interaction prediction model based on multi-source biological interaction data, IIMCCMA. This model first uses the Gaussian kernel function to calculate the GIP kernel similarity of circRNA and the GIP kernel of miRNA based on the circRNA-miRNA interaction network. Then, on the basis of the circRNA-cancer interaction network and the miRNA-cancer interaction network, the cosine function is used to calculate the functional similarity of circRNA and miRNA, respectively. Afterward, the different similarities of circRNAs and the different similarities of miRNAs were integrated separately. The known circRNA-miRNA interaction network is used to construct a heterogeneous network for extracting topological features of circRNA and miRNA, and the network embedding algorithm (NetMF) is used to obtain the low-dimensional space vectors of circRNA and miRNA, respectively. Finally, based on the fusion features, an improved inductive matrix completion algorithm is used to predict the potential interaction between circRNA and miRNA. In order to test the performance of the IIMCCMA, this paper selects four circRNA-disease potential interaction prediction models for comparison. The 10-fold cross-validation results show that compared with the other four models, the IIMCCMA achieved higher AUROC and AUPR values. Therefore, it is proved that IIMCCMA has better predictive ability. Moreover, the results of a case study based on miRNA miR-1455p show that the IIMCCMA model can correctly identify the potential interaction between circRNA and miRNA.

Although, the IIMCCMA model has shown excellent performance in predicting the potential interaction between circRNA and miRNA. However, there are still some shortcomings and limitations. (1) The imbalance of positive and negative samples in interaction data. Because the efficiency of identifying circRNA and miRNA interactions through biological experiments is low, in the existing circRNA-miRNA interaction network, the experimentally verified interactions are far less than the unknown interactions. The sparse circRNA-miRNA interaction network greatly affects the performance of the prediction model (Lan et al., 2016b, 2020b; Lei et al., 2020). Therefore, in the follow-up work, we will try to pre-fill the original interaction matrix to alleviate the sparsity of the known interaction network and enhance the performance of the model. (2) Parameter setting. There are a certain number of parameters in the IIMCCMA model that need to be set manually. The quality of the parameters needs to be confirmed through experimental verification. In addition, too many parameters will reduce the learning and generalization capabilities of the model. Therefore, no parameter or self-learning parameter model will be the main work in the future (Lan et al., 2016a, 2017, 2022b; Chen et al., 2021).

## Data availability statement

The original contributions presented in the study are included in the article/supplementary material, further inquiries can be directed to the corresponding author.

## Author contributions

SY designed the work. DY and LN performed all the experiments. DY, LN, MQ, and SW wrote the manuscript. All authors listed have made a substantial, direct, and intellectual contribution to the work and approved it for publication.

## Conflict of interest

The authors declare that the research was conducted in the absence of any commercial or financial relationships that could be construed as a potential conflict of interest.

## Publisher’s note

All claims expressed in this article are solely those of the authors and do not necessarily represent those of their affiliated organizations, or those of the publisher, the editors and the reviewers. Any product that may be evaluated in this article, or claim that may be made by its manufacturer, is not guaranteed or endorsed by the publisher.
